# A Unique Assemblage of Engraved Plaquettes from Ein Qashish South, Jezreel Valley, Israel: Figurative and Non-Figurative Symbols of Late Pleistocene Hunters-Gatherers in the Levant

**DOI:** 10.1371/journal.pone.0160687

**Published:** 2016-08-24

**Authors:** Alla Yaroshevich, Ofer Bar-Yosef, Elisabeta Boaretto, Valentina Caracuta, Noam Greenbaum, Naomi Porat, Joel Roskin

**Affiliations:** 1 Israel Antiquities Authority, Jerusalem, Israel; 2 Harvard University, Department of Anthropology, Peabody Museum of Archeology and Ethnology, Cambridge, Massachusetts, United States of America; 3 Max Planck-Weizmann Institute Center for Integrative Archaeology, DREAMS Radiocarbon Laboratory, Weizmann Institute of Science, Rehovot, Israel; 4 Department of Geography and Environmental Studies, University of Haifa, Haifa, Israel; 5 Geological Survey of Israel, Jerusalem, Israel; 6 Department of Maritime Civilizations, Charney School of Marine Studies and the Leon Recanati Institute for Maritime Studies (RIMS), University of Haifa, Haifa, Israel; 7 Department of Marine GeoSciences, Charney School of Marine Studies, University of Haifa, Haifa, Israel; 8 School of Sciences, Achva Academic College, Beer Tuvya, Israel; Universidade do Algarve, PORTUGAL

## Abstract

Three engraved limestone plaquettes from the recently excavated Epipaleolithic open-air site Ein Qashish South in the Jezreel Valley, Israel comprise unique evidence for symbolic behavior of Late Pleistocene foragers in the Levant. The engravings, uncovered in Kebaran and Geometric Kebaran deposits (*ca*. 23ka and *ca*. 16.5ka BP), include the image of a bird—the first figurative representation known so far from a pre-Natufian Epipaleolithic—along with geometric motifs such as chevrons, crosshatchings and ladders. Some of the engravings closely resemble roughly contemporary European finds interpreted as "systems of notations" or "artificial memory systems"–records related to timing of seasonal resources and associated aggregation events of nomadic groups. Moreover, similarly looking signs and patterns are well known from the context of the local Natufian—a final Epipaleolithic culture of sedentary or semi-sedentary foragers who started practicing agriculture. The investigation of the engravings found in Ein Qashish South involves conceptualizations developed in studies of European and local parallels, a selection of ethnographic examples and preliminary microscopic observations of the plaquettes. This shows that the figurative and non-figurative images comprise a coherent assemblage of symbols that might have been applied in order to store, share and transmit information related to social and subsistence realms of mobile bands. It further suggests that the site functioned as a locality of groups' aggregation and indicates social complexity of pre-Natufian foragers in the Levant. While alterations in social and subsistence strategies can explain the varying frequency of image use characterizing different areas of the Late Pleistocene world—the apparent similarity in graphics and the mode of their application support the possibility that symbol-mediated behavior has a common and much earlier origin.

## Introduction

The meaning, purpose and circumstances of the emergence of figurative and non-figurative art have been studied for a long time based mainly on ornate caves and numerous mobile objects uncovered in Upper Paleolithic (UP) contexts in Western Europe (see [1] for a review). Elaborate rock paintings from the Indonesian island of Solawesi—recently dated ca 35–40 ka BP—indicate contemporaneity with the earliest European parallels and imply a much older origin of artistic manifestations [2]. Such possibility suggests that the use of both figurative and non-figurative images and signs comprised an integral part of behavior of modern humans who left the African continent a few tens of millennia earlier [2, 3]. A variety of artefacts with symbolic connotations, including different engraved patterns recovered in Middle Stone Age (MSA) contexts in South Africa, comprise a substantial base for this hypothesis [4–9].

In this regard the extreme rarity of artistic expressions left by mobile hunters-gatherers in Southwest Asia and particularly in the Levant—the "corridor" passed by groups migrating out of Africa—is puzzling [10, 11]. Notwithstanding the extensive research, no cave paintings have so far been recorded here, while mobile art in appreciable numbers does not appear before the emergence of the Natufian (*ca*. 15–11.5 cal BP)–a final Epipaleolithic culture of sedentary or semi-sedentary forbearers of Neolithic agriculturalists [12, 13]. Pre-Natufian Epipaleolithic contexts in the Levant yielded so far only a few decorated items. These include a limestone pebble with ladder and crosshatching patterns from Urkan a-Rub in the Jordan Valley [14]; a fragmented stone plaquette with a repetitive design of rectangles from Kharaneh IV in Jordan [15]; and a recently published incised stone with a design resembling the ladder motif from yet another Jordan site, at Madamagh rockshelter [16]. Simple linear designs engraved on bone implements reported from Ohalo II (Israel), Jiita II and Ksar Akil layer 8c (both in Lebanon) as well as from Kharane IV (Jordan) complete this modest assemblage [17, 18, 19]. The only figurative image found so far in the region, an engraving of a horse from Hayonim Cave, Israel [20], is affiliated with the Upper Paleolithic Aurignacian.

Against this background, the three engraved limestone plaquettes, i.e., the ladder plaquette, the bird plaquette and the chevron plaquette from Ein Qashish South (EQS), an Epipaleolithic open-air camp site in the Jezreel Valley, comprise a unique find. Based on stratigraphy, radiocarbon assays and associated flint tools, the items are attributed to the Kebaran and Geometric Kebaran entities, and dated to *ca*. 23 and *ca*. 16.5ka BP. The image of a bird displayed on one of the plaquettes, comprises the first figurative representation from Epipaleolithic context predating Natufian culture of sedentary or semi-sedentary foragers who started practicing agriculture. Other engravings represent geometric motifs, namely, cross-hatching, chevron and ladder, the latter closely resembling roughly contemporary and later Levantine and European examples interpreted by some authors as system of notations [20–25] or artificial memory systems [6, 26, 27].

The EQS engravings, exceptional in terms of number and variability of the motifs, provide a unique source for investigating symbolic behavior of mobile groups occupying the Levant during the Late Pleistocene. Our attempts to interpret the findings involve archaeological correlates from European contexts, local parallels known mostly from the Final Epipaleolithic Natufian as well as some ethnographic evidence. The interpretation reflects on the function of the site and on social and subsistence strategies practiced by the Late Pleistocene foragers in the Levant. We further briefly discuss the possible explanations for apparent similarity in graphic symbols deriving from distinctly remote areas of the late Pleistocene world as well as the role of the social and subsistence strategies in the varying frequency of image use.

## Materials and Methods

### The site and its surroundings

**Fig 1:**
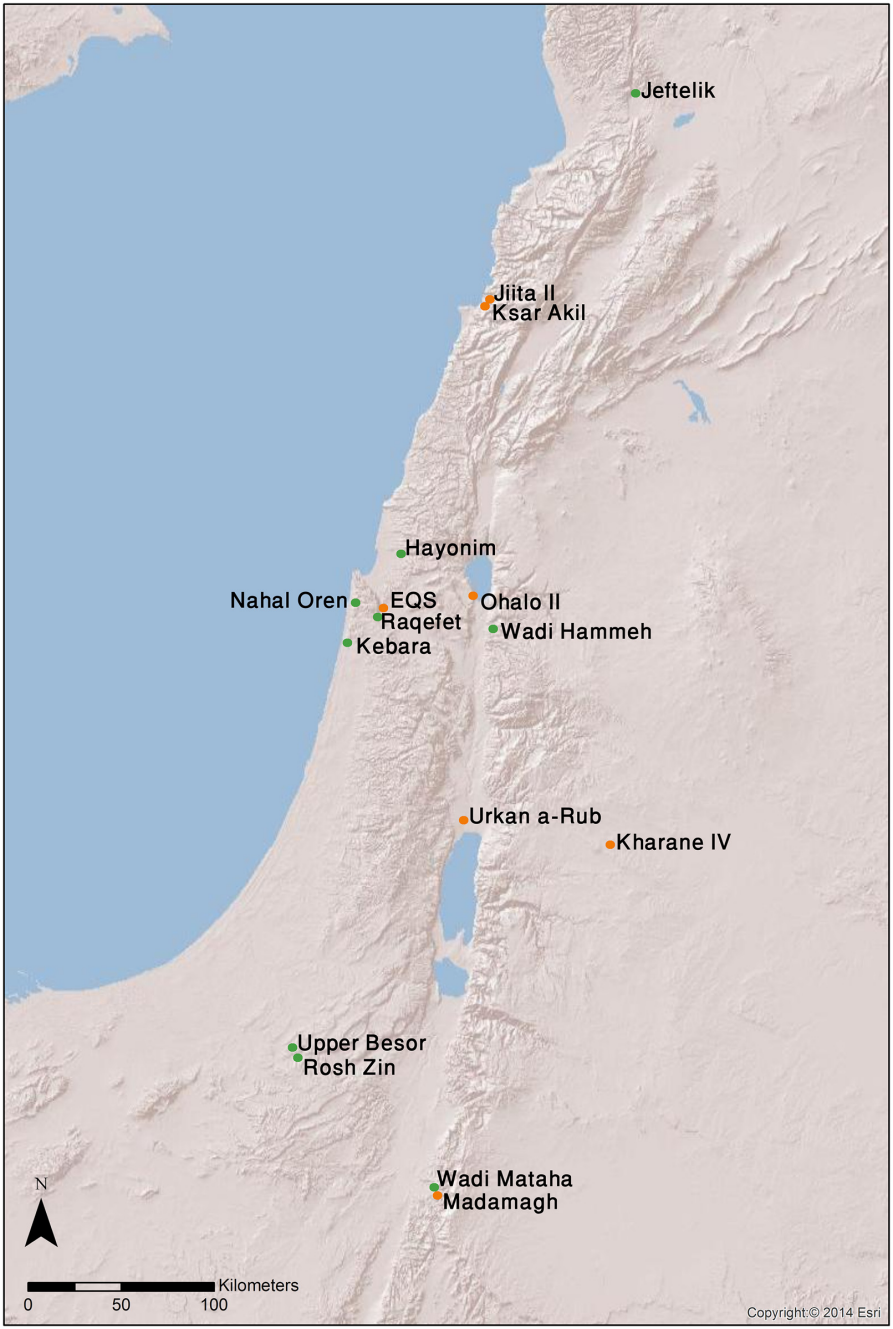
Location of Ein Qashish South and other Levantine sites where examples of graphic imagery similar to EQS were found. Orange signs indicate Kebaran, Geometric Kebaran and Late UP contexts; green signs indicate Natufian contexts.

**Fig 2:**
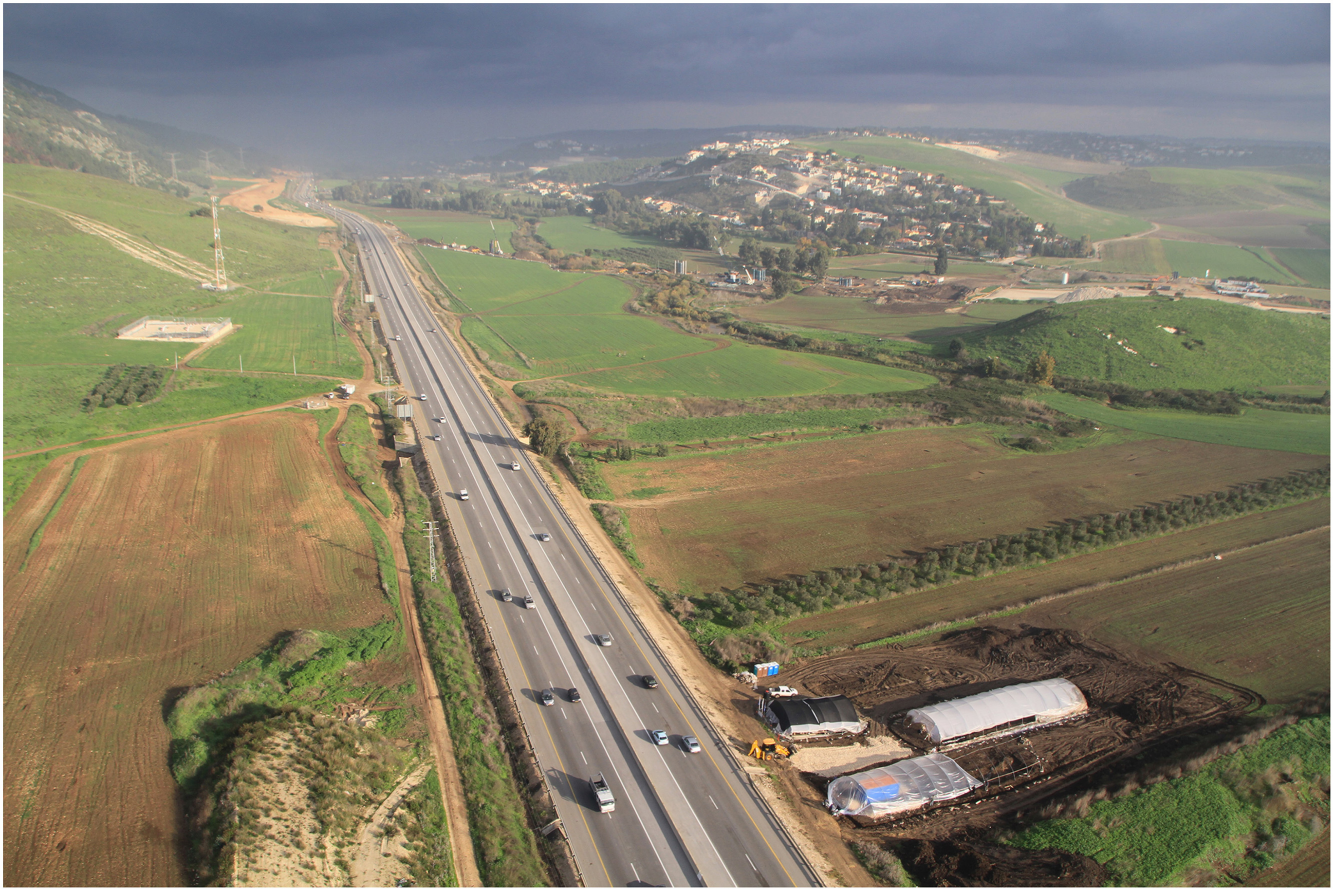
Ein Qashish South, looking north. Tel Qashish, bound by the Qishon stream, appears to the north of the present excavation. The foothills of the Carmel Mount are in the west.

EQS (geographic coordinates: 32°40'55.50N, 35°6'24.31E) is located in the Jezreel Valley, Israel, *ca*. 500m east of Mount Carmel and about 300 m south of the eponymous spring situated at the piedmont of Tel Qashish, a small mound on a hillock containing mostly Bronze Age deposits (Figs 1 and 2). The perennial Qishon River flows some 200 m east-north of the site and debouches into the Mediterranean Sea in the bay of Haifa [28].

EQS was found during test trenching performed prior to the construction of a highway segment; following salvage excavations took place in the winter of 2012–2013, initially in five distinct sub-areas marked A-E. Epipaleolithic *in situ* deposits were found in Areas D and E; these two areas were subsequently extended into Areas D and G (northern area) and Areas E and F (southern area), each covering *ca*. 120m^2^. During the winter excavations, greenhouses against the rain protected each area separately, thus leaving an unexcavated space in between.

The excavations, undertaken on behalf of the Israel Antiquities Authority (permit A-6655) and underwritten by the Road # 6 construction company were directed by the first author. The three engraved plaquettes as well as the rest of the artefacts presented in the paper can be accessed at the collections of the Israel Antiquities Authority, Ha-Marpea st., Har Hotzvim, Jerusalem.

### Stratigraphy

**Fig 3:**
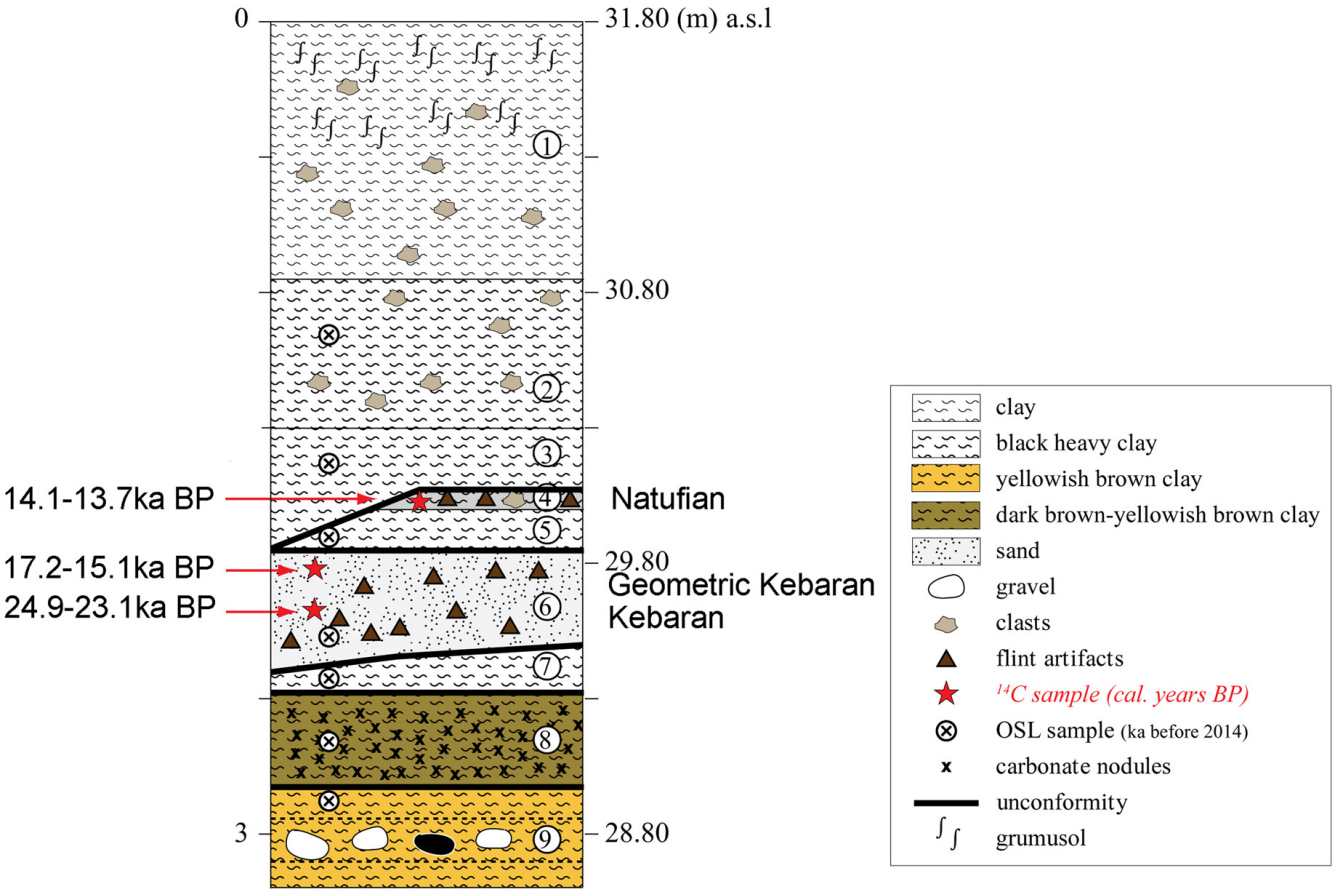
Stratigraphy and chronology of EQS.

**Fig 4:**
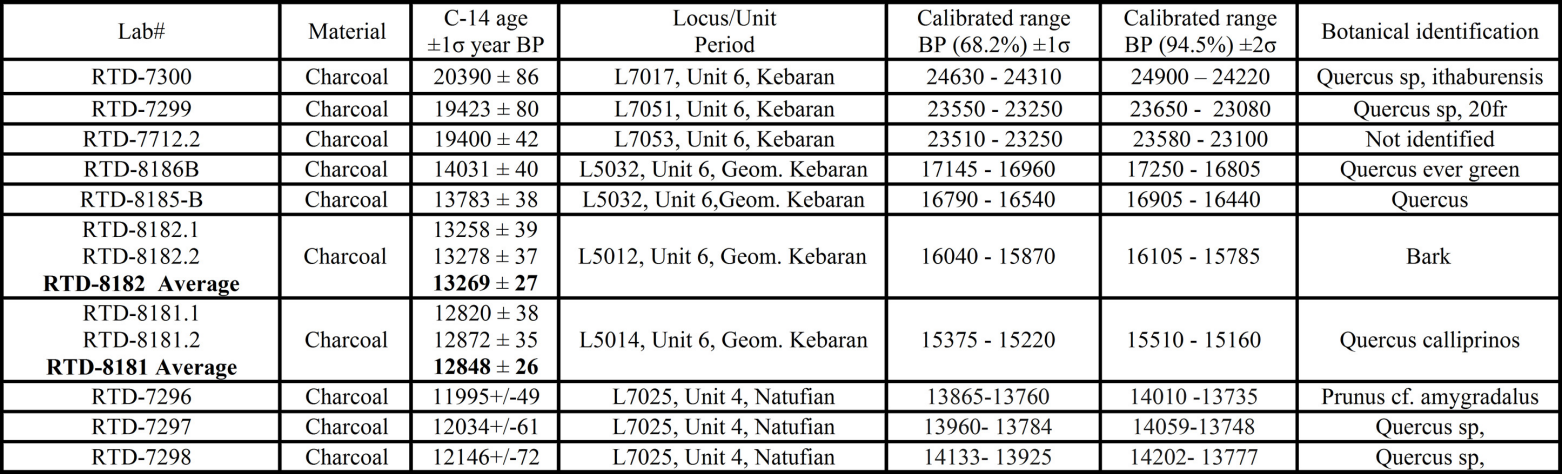
Radiocarbon readings of EQS. ^14^C ages are reported in conventional radiocarbon years (before present = 1950) in accordance with international convention [29]. Thus all calculated ^14^C ages have been corrected for the fractionation so as to refer the results to be equivalent with the standard δ_13_^13^C value of -25% (wood). Calibrated ages in calendar years have been obtained from the calibration tables [30] by means of OxCal v. 4.2 [31–33].

The stratigraphy of Ein Qashish South (EQS) comprises nine sedimentary units (Fig 3). The depositional, mostly fluvial and marshy environment alternated with periods of relative dry spells of varying duration. Most of the sedimentary accumulations originated from two sources. The clayey sediments were deposited during flooding of the Kishon River in times of greater winter precipitation. The gravels were mostly washed in from the slopes of Mount Carmel that are steeply rising from about 100 meters to 450–500 m above sea level west of the site. In situ Epipaleolithic occupations were exposed in Unit 4 (Natufian) and Unit 6 (Kebaran and Geometric Kebaran). Their chronology derived from a total 12 calibrated radiocarbon assays (Fig 4).

**Fig 5:**
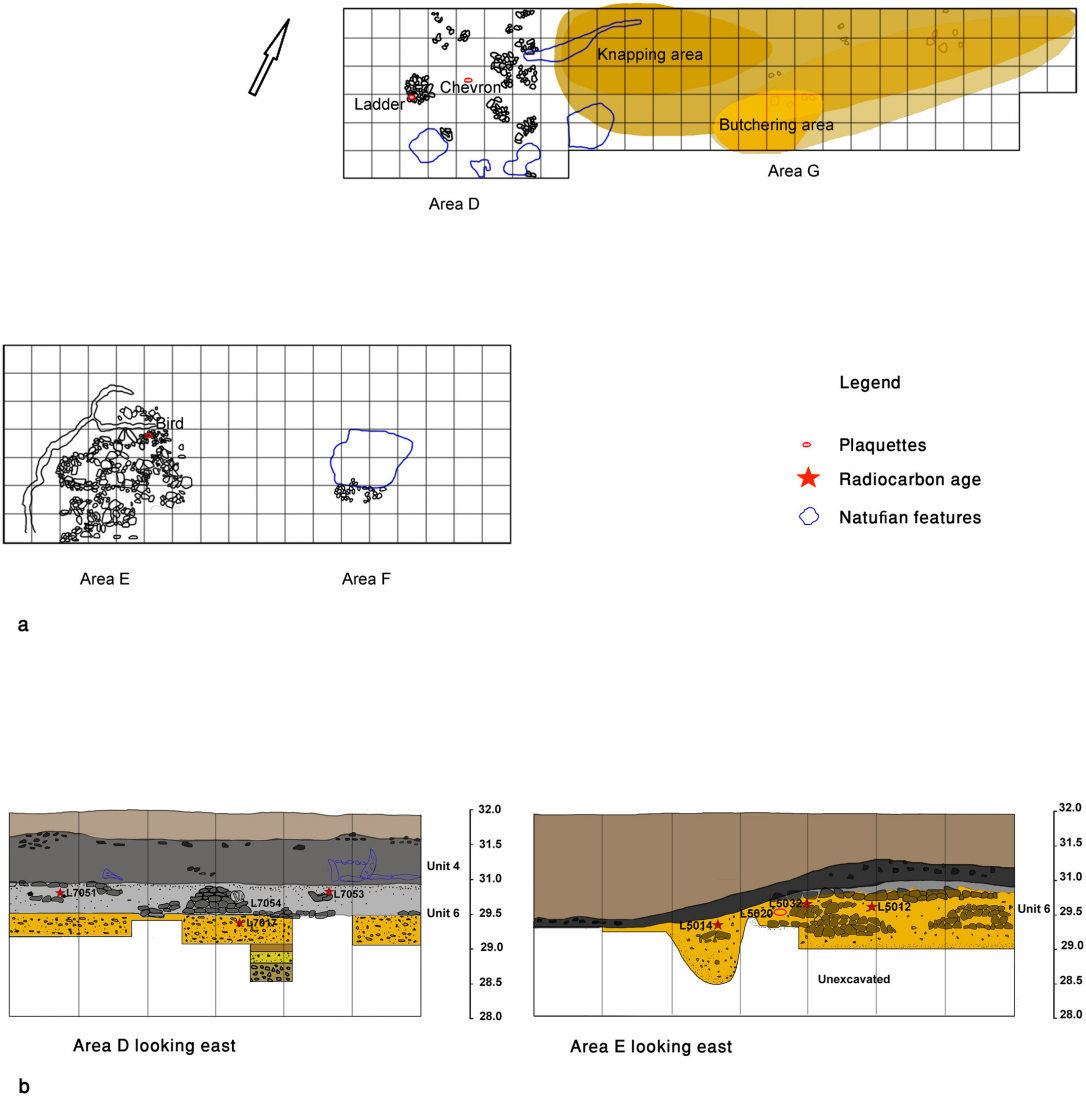
Schematic plan and composite sections of Ein Qashish South indicating find position of the plaquettes and the main loci mentioned in the text. (a) Plan of the site representing main Kebaran and Geometric Kebaran features (Unit 6). Natufian features (Unit 4) are indicated by outlines. (b) Composite sections of the Areas D and E, looking east.

Unit 1: Dark brown, massive clay, with carbonate nodules and occasional clasts and roots- a grumosol (vertisol).
Unit 2: Very dark brown, heavy, massive, compacted clay, with a few large (up to cobble-stone sized) clasts, and slickensides.
Unit 3: Dark brown-black, compacted, hard, massive clay with slickensides, which unconformably overlies Unit 4. It contains up to 3 cm size carbonate nodules with voids, gypsum concretions and some gravel. At the base it is mottled by yellowish iron concretions and stains indicating reduction processes in a water-saturated environment. The unit probably represents a temporary standing water body/marsh.
Unit 4: Black-very dark brown, massive, heavy clay, with some gravel, unconformably overlies Unit 5. The unit contains a number of well-defined features affiliated with the Natufian culture, including stone built facilities that incorporate horns of wild cattle, as well as lenses of a distinctive brown sandy sediments (Fig 5a and 5b). The features were found in the northern area (Areas D and G) and in the eastern part of the southern area (Area F). The samples of charcoal submitted for ^14^C analyses provide a time range between 14.2 and 13.7ka cal BP (Fig 4).
Unit 5: Black-very dark brown, heavy clay, with some gravel which unconformably overlies Unit 6. The structure is prismatic with slickensides and is archaeologically virtually sterile.
Unit 6: Sand and clay unit that unconformably overlies Unit 7. The top is composed of grayish clayey-sand, whereas the base and middle part are composed of massive, friable-slightly hard sand, pebbles and cobbles of limestone and basalt. The unit contains flint artifacts, chipped limestone items, grinding tools, fragments of bones and charcoal. Three charcoal samples obtained from the western part of the northern area (Areas D) gave ages ranging from 24.9 to 23.1ka cal. BP (Figs 4 and 5b). The samples from the eastern part of the southern area (Area E) yielded five radiocarbon readings ranging between 17.2 and 15.2ka cal. BP (Figs 4 and 5b).
Unit 7: Dark brown, massive, heavy clay unit, with slickensides, which unconformably overlies Unit 8.
Unit 8: Dark brown, massive, compacted clay unit, which unconformably overlies Unit 9. It contains 40% carbonate and gypsum concretions up to 0.5 cm in size, possibly related to high groundwater level and few coated chalk, marl and chert gravel 2–3 cm in size.
Unit 9: Alternating, thin yellowish-brown clay layers (10 cm thick) with matrix-supported gravel layers 20–30 cm thick that contains about 40% gravel with yellowish-brown clay matrix. The gravel is coarse, consisting of angular to sub-rounded pebbles and cobbles of limestone, chert and dolomite, up to 12 cm in size. The unit is mottled and includes manganese stains and concretions. The unit was water-saturated, indicating ground water level at present as well as in the past.

## The find context of the engraved plaquettes

**Fig 6:**
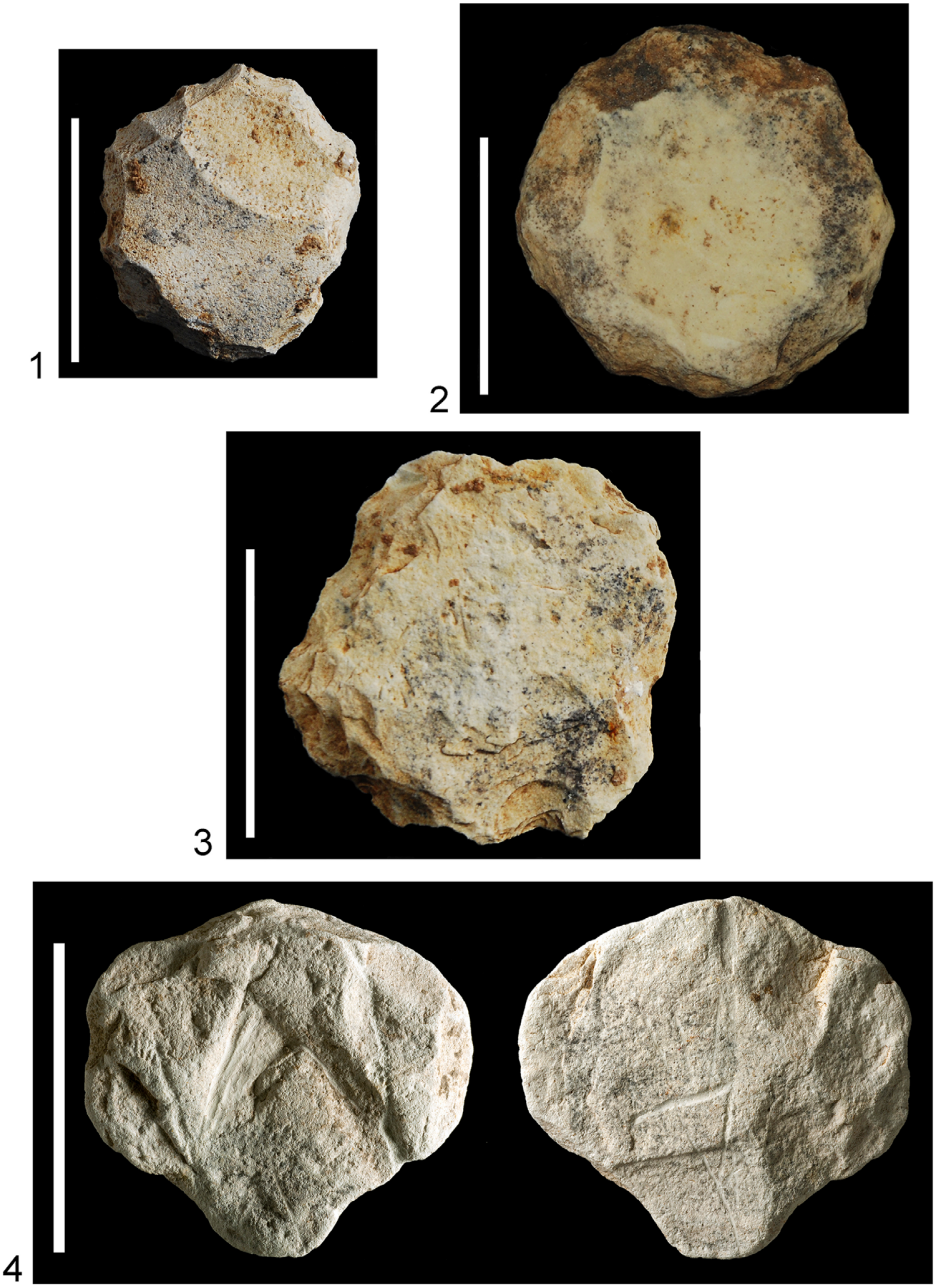
Limestone industry characteristic of Unit 6. 1–3: endscrapers; 4: plaquette with initiated engraving, found in the same basket as the bird plaquette. The scale is 5cm in each case.

**Fig 7:**
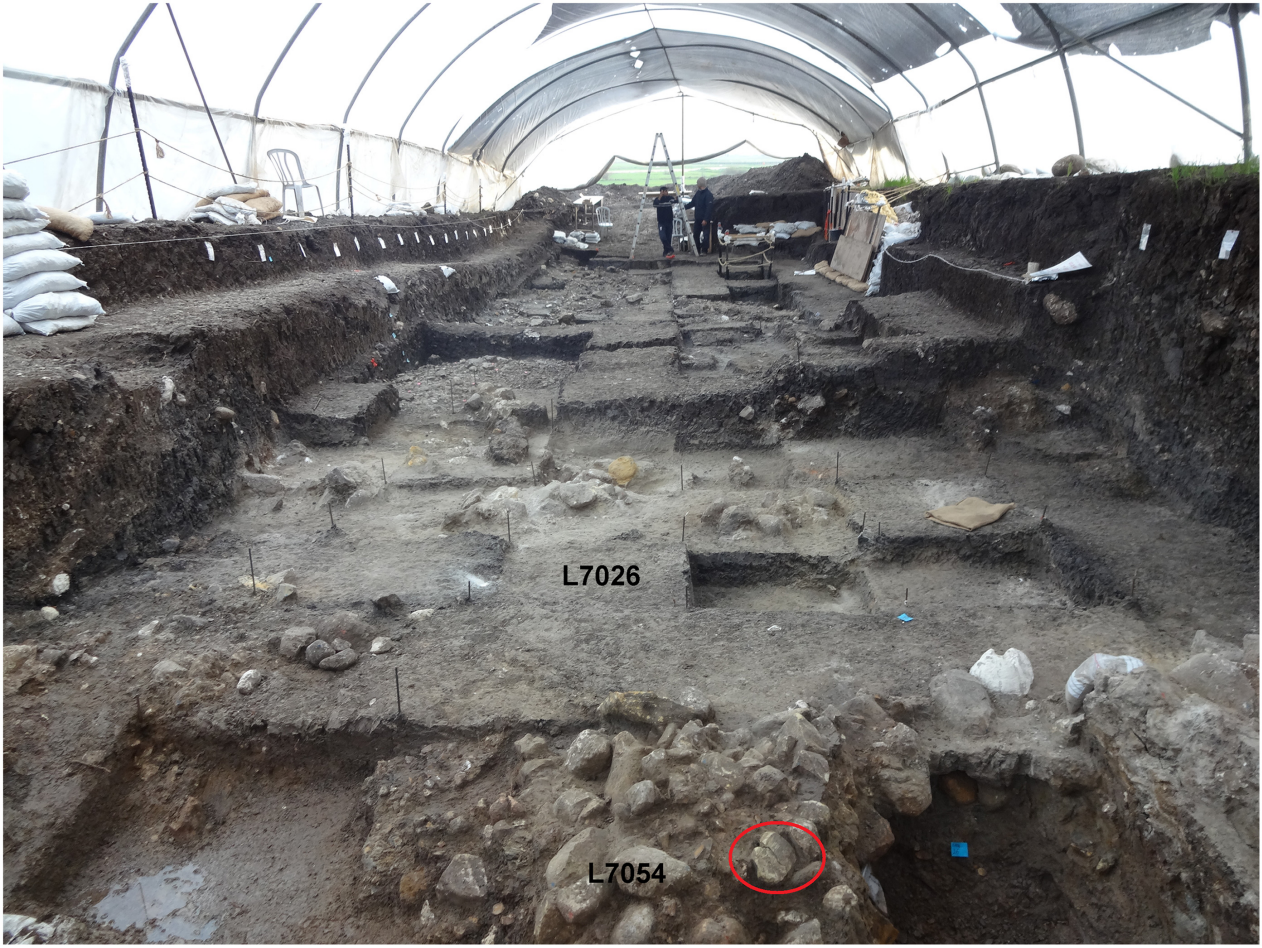
Northern area (Areas D and G), looking east. In the foreground the ladder plaquette is shown *in situ* incorporated into accumulation of stones (L7054). The original find position of the chevron plaquette is indicated (L7026).

**Fig 8:**
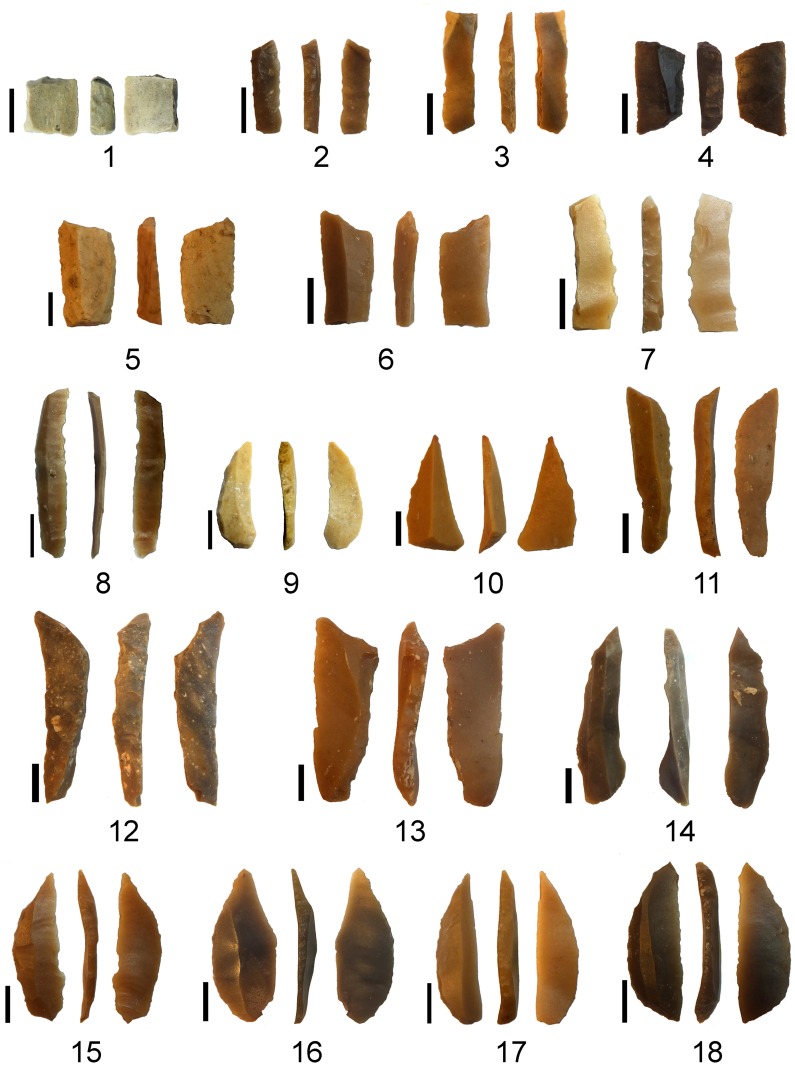
Microlithic tools found in association with the plaquettes. 1–8: trapeze/rectangles; 9, 15–18: arch-backed bladelets; 10, 14: truncated bladelets; 11–13: backed and truncated bladelets (Kebara points). 1, 2, 6–8: L5020; 3–5, 12–13: L5012; 9–10: L7026; 11, 14–15: L7054; 16–18: L7051. The scale is 5mm in each case.

All three engraved plaquettes (ladder, bird and chevron) were found in Unit 6. The plaquettes *per se* are part and parcel of the prominent limestone industry characteristic of this unit which include chipped and ground stone tools (Fig 6: 1–3). The ladder plaquette (see below) was found incorporated in the largest of the stone installations (L7054), exposed in the western part of the northern area (Area D, Figs 5 and 7). The chevron plaquette was found during sieving of the sandy matrix deriving from a spot about 1.5m east of this installation (L7026, Figs 5 and 7). The microlithic assemblages derived from different loci in the area, and in particular from L7054 and L7026, are dominated by non-geometric forms such as arch-backed bladelets (Fig 8: 15–18), truncated bladelets (Fig 8: 10, 14) and Kebara points (Fig 8: 11–13). These corroborate the radiocarbon readings in the area affiliating the findings to the Kebaran culture [34–35].

**Fig 9:**
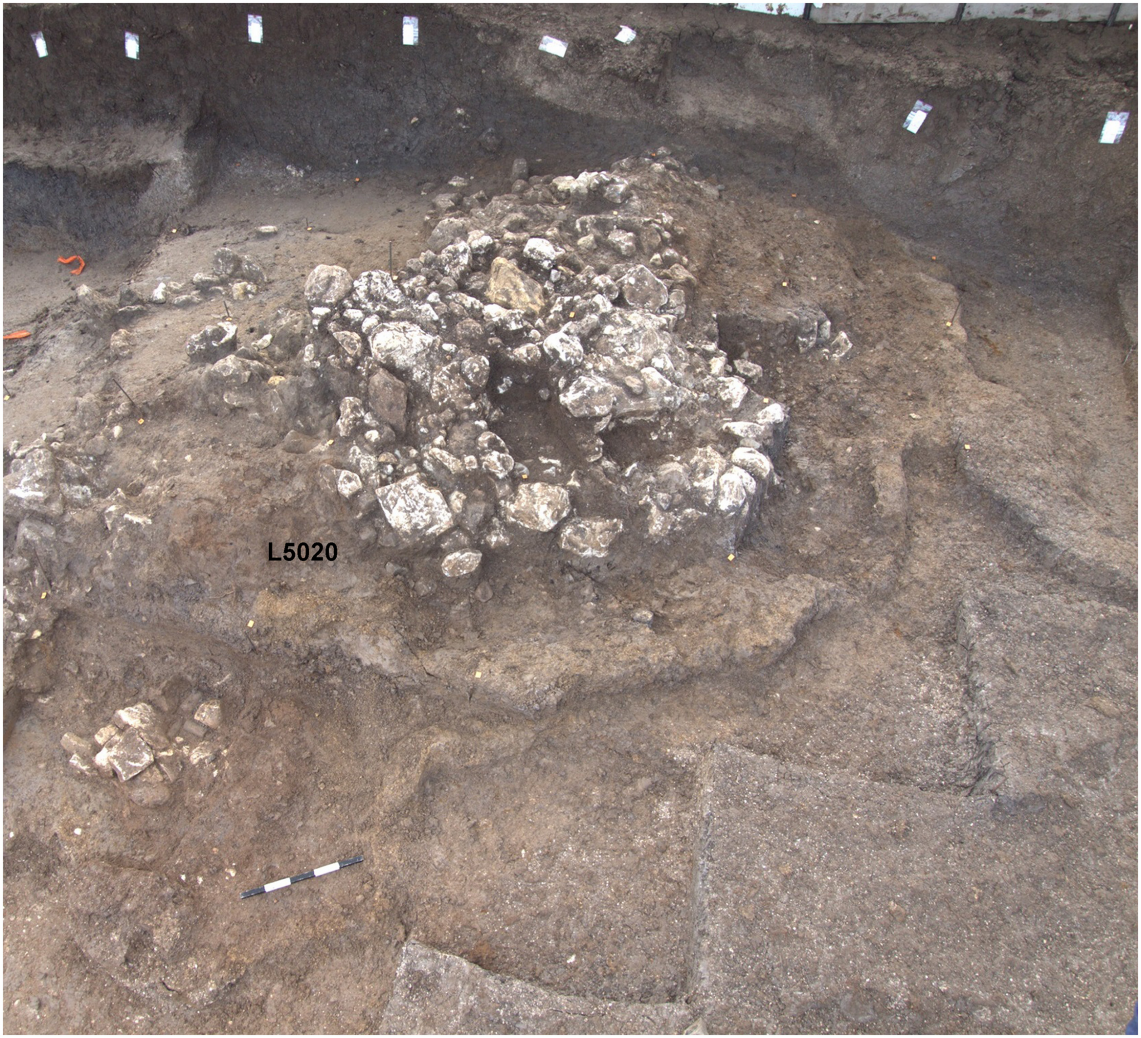
Southern area (Areas E and F), looking south-east. The location of the bird plaquette is indicated (L5020).

The bird plaquette was found in the western part of the southern area (Area E) in the sandy sediment (L5020) within a complex of stone installations enclosed by a ridge of distinctive clayish sediment and interpreted as remains of a hut-like structure (Figs 5 and 9). Notably, in the same basket another plaquette was found, bearing a few engraved lines, probably a pattern which had just been initiated (Fig 6:4). The radiocarbon dates obtained from the adjacent stone installation (L5032) allow a time estimate for the plaquette around 17–16.5ka BP (Fig 4), i.e., fitting the Geometric Kebaran entity [34, 35]. These adjacent loci as well as the loci above them are characterized by high frequencies of trapeze/rectangles (Fig 8: 1–8) corroborating the affiliation of the plaquette to this cultural entity.

### The engraved plaquettes

#### The bird plaquette

**Fig 10:**
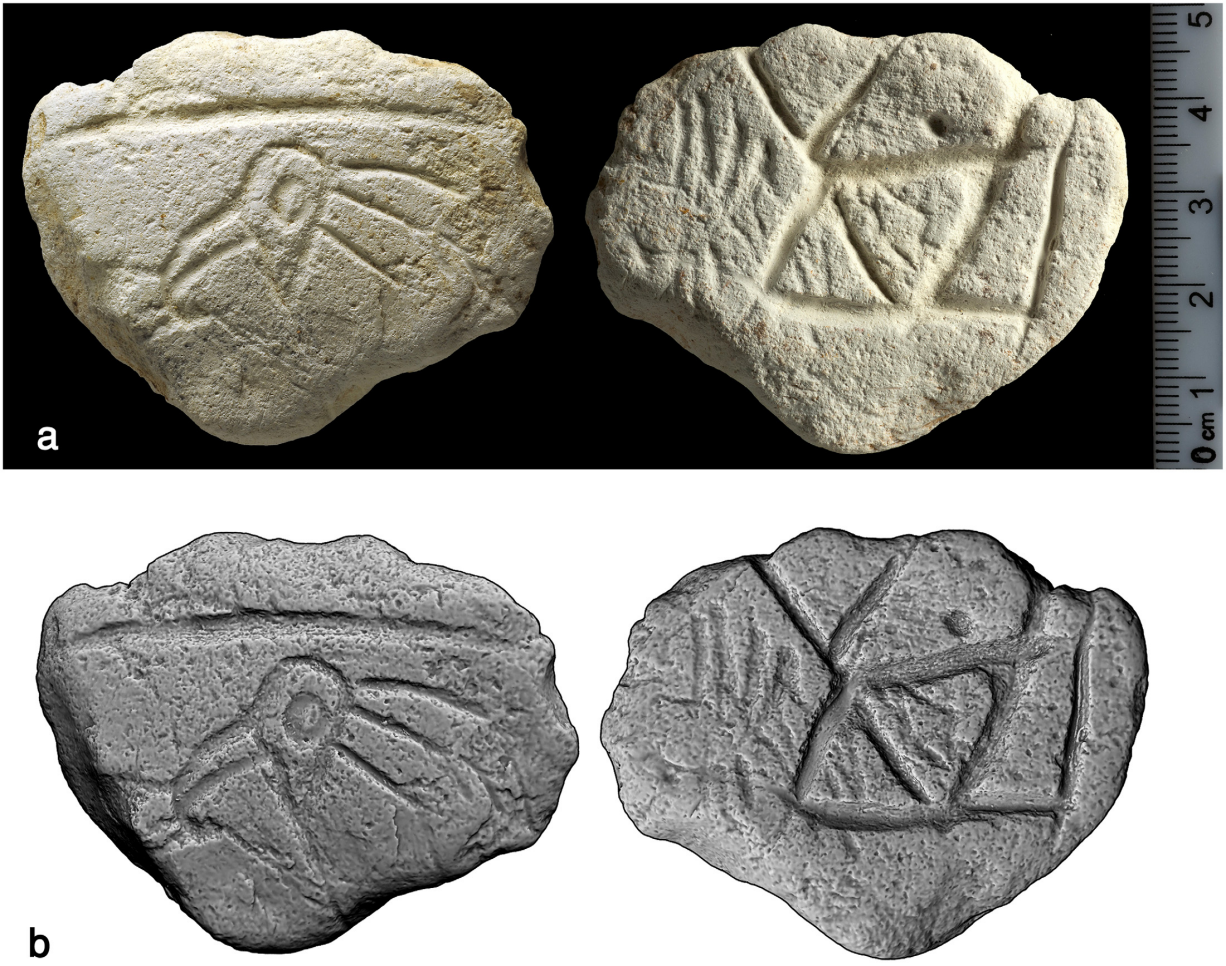
The bird plaquette. Above: photograph, reprinted from [28] under a CC BY license, with permission from [Antiquity], original copyright [2014].” Below: 3D scanning.

**Fig 11:**
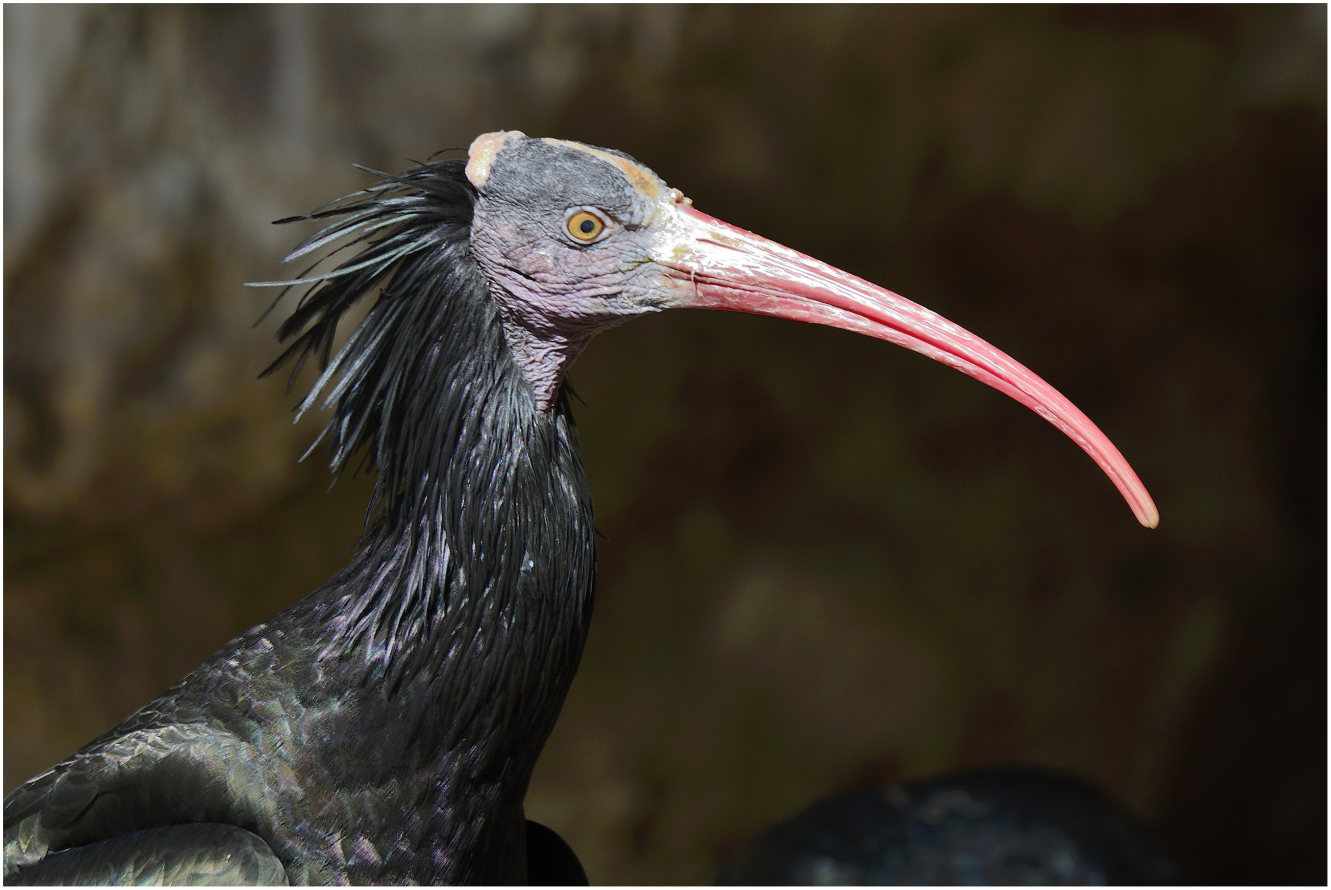
Bald Ibis. By courtesy of Daniel Berkowic, Department of Zoology, Tel Aviv University, Natural History Collections.

A roughly oval, thin plaquette with irregular margins, measuring 58 x 44 x 7mm, had been incised on both sides (Fig 10). A slightly convex recto represents the head of a bird seen in profile, together with a slightly curved, deeply incised line right above it. The bird is characterized by a large, curved beak and three “feathers” in the form of little curvilinear, roughly parallel lines attached to the bird’s nape. A large round eye appears in the middle of the upturned, drop-shaped head. The image has been independently identified by two ornithologists as that of a bald ibis (*Geronticus eremita)*–a migratory species, overall black with red head, beak and legs (Fig 11). Critically endangered today, the bald ibis was once widespread across South-West Asia, North Africa as well as Southern and Central Europe [36]. Short lines obliquely attached to the lower part of the bird's feathers are notable. These closely resemble the "cues" observed on the EQS ladder pattern as well (see below).

The slightly concave verso of the bird plaquette contains a motif composed of several deeply incised chevron-like signs, two of them forming a rhombus divided into two triangles in the middle of the composition. A small, non-perforated hole can be seen within one of the chevrons. The lines creating some of the chevrons cross each other slightly at their point of convergence. Another characteristic feature of the motif is a roughly straight line engraved close to the right lateral edge of the plaquette, adjacent to the chevrons. The entire design appears homogenous in terms of both the relatively considerable width and depth of the engraved lines. The chevron motif overlays a previously incised pattern which included two series of curvilinear, roughly parallel lines resembling the feathers of the bird on the recto of the plaquette. It seems that a small rhombus observed within a bigger one in the middle of the composition also belongs to this previously engraved pattern. Very small red stains, probably of ochre, can be noticed on both sides of the plaquette (Fig 10).

#### The chevron plaquette

**Fig 12:**
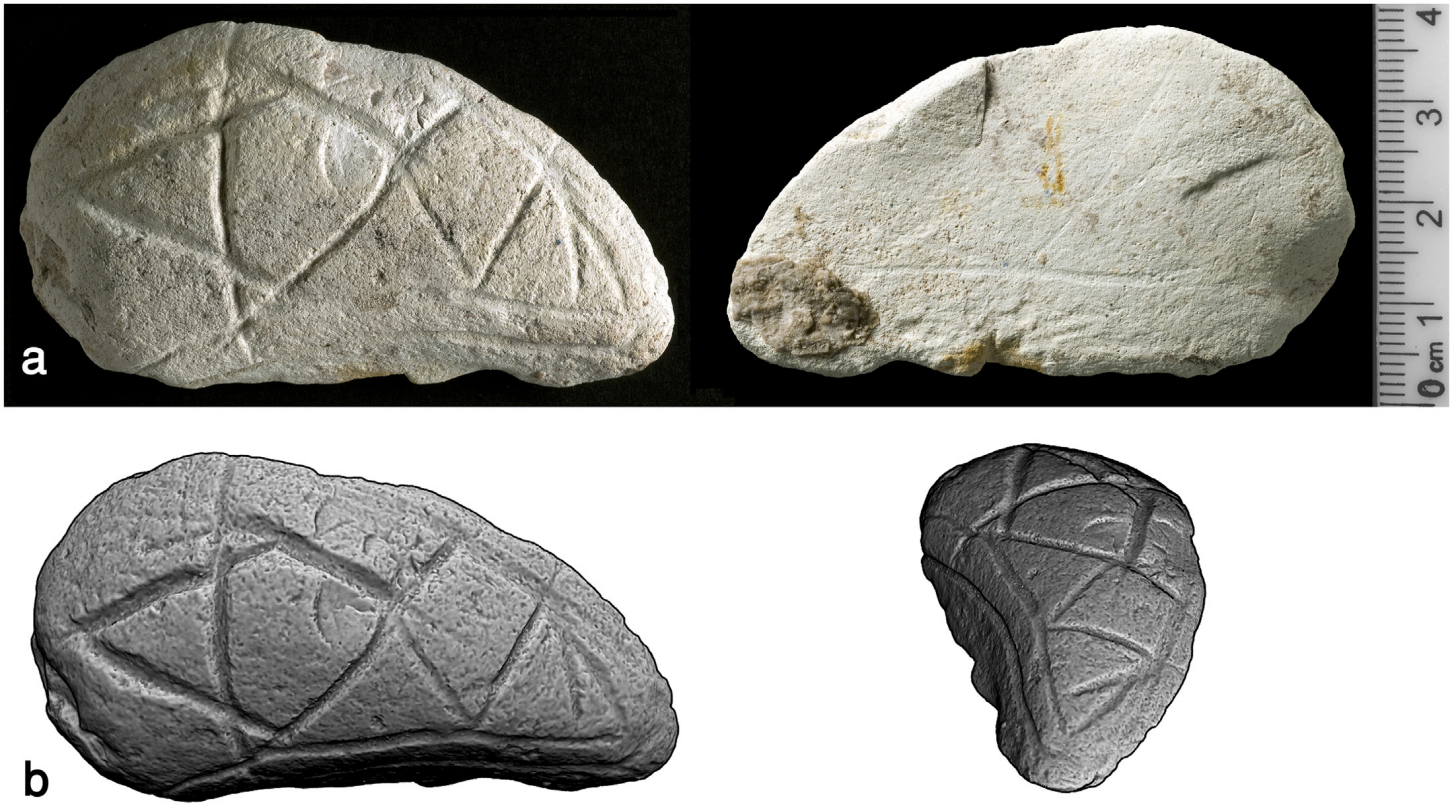
The chevron plaquette.

A sub-triangular flake, measuring 63 x 33 x 8mm, bears engraving only on its convex dorsal surface (Fig 12). The engraved motif resembles the verso of the bird plaquette in several aspects. First, in both cases chevrons or V-shaped incisions are the main component of the composition; second, some of the chevrons cross each other while others create rhombus-like figures; third, in both compositions roughly rectilinear lines are engraved along the plaquette's margin, adjacent to the chevron motif. Another shared characteristic is the execution of the incisions, appearing rather similar in both width and depth. Possibly the ventral, flat side of the flake was also intended to be incised, but for unknown reasons was never executed.

#### The ladder plaquette

**Fig 13:**
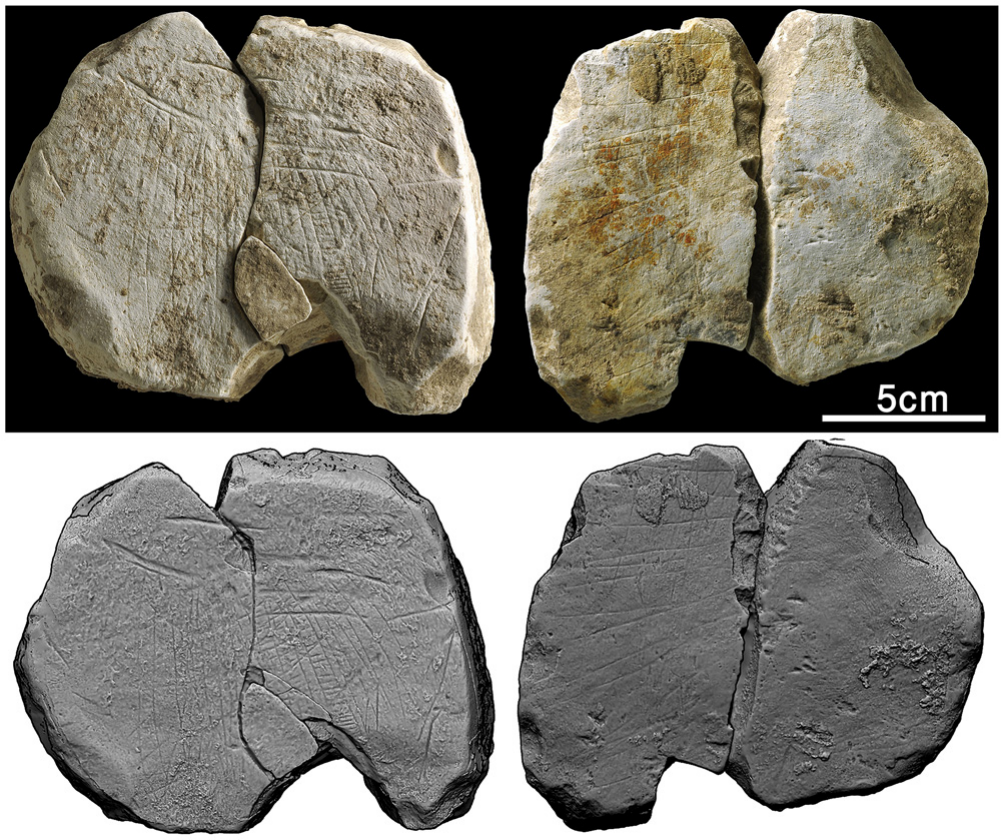
The ladder plaquette. Above: photograph, reprinted from [28] under a CC BY license, with permission from Antiquity journal, original copyright [28].” Below: 3D scanning.

This plaquette is roughly round in shape (120 x 145 x 25mm), smoothed by grinding on both sides as well as along the edges (Fig 13). The plaquette was found *in situ*, broken into two nearly equal pieces, but still holding together, i.e., in articulation, indicating that the plaquette was abandoned and placed on the accumulation of stones (L7054) when still complete, probably when it was still only cracked (Fig 7). Missing are two sub-triangular fragments, indicating ancient breaks. The plaquette is engraved on both sides.

**Fig 14:**
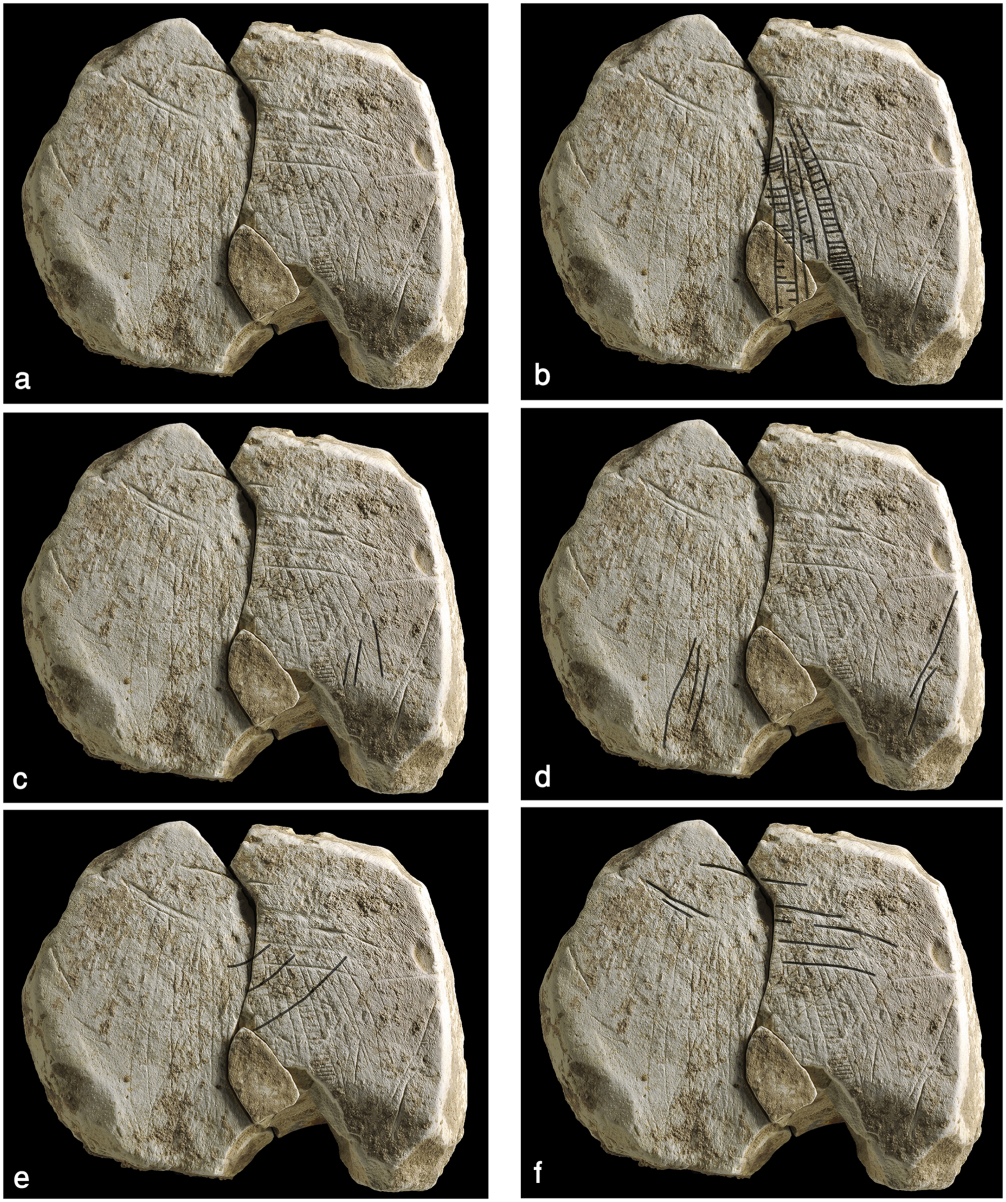
The components of the ladder motif. (a) Ladder motif. (b) "Ladders". (c) "Cues". (d) Long oblique lines. (e) Narrow crossing lines. (f) Broad crossing lines.

**Fig 15:**
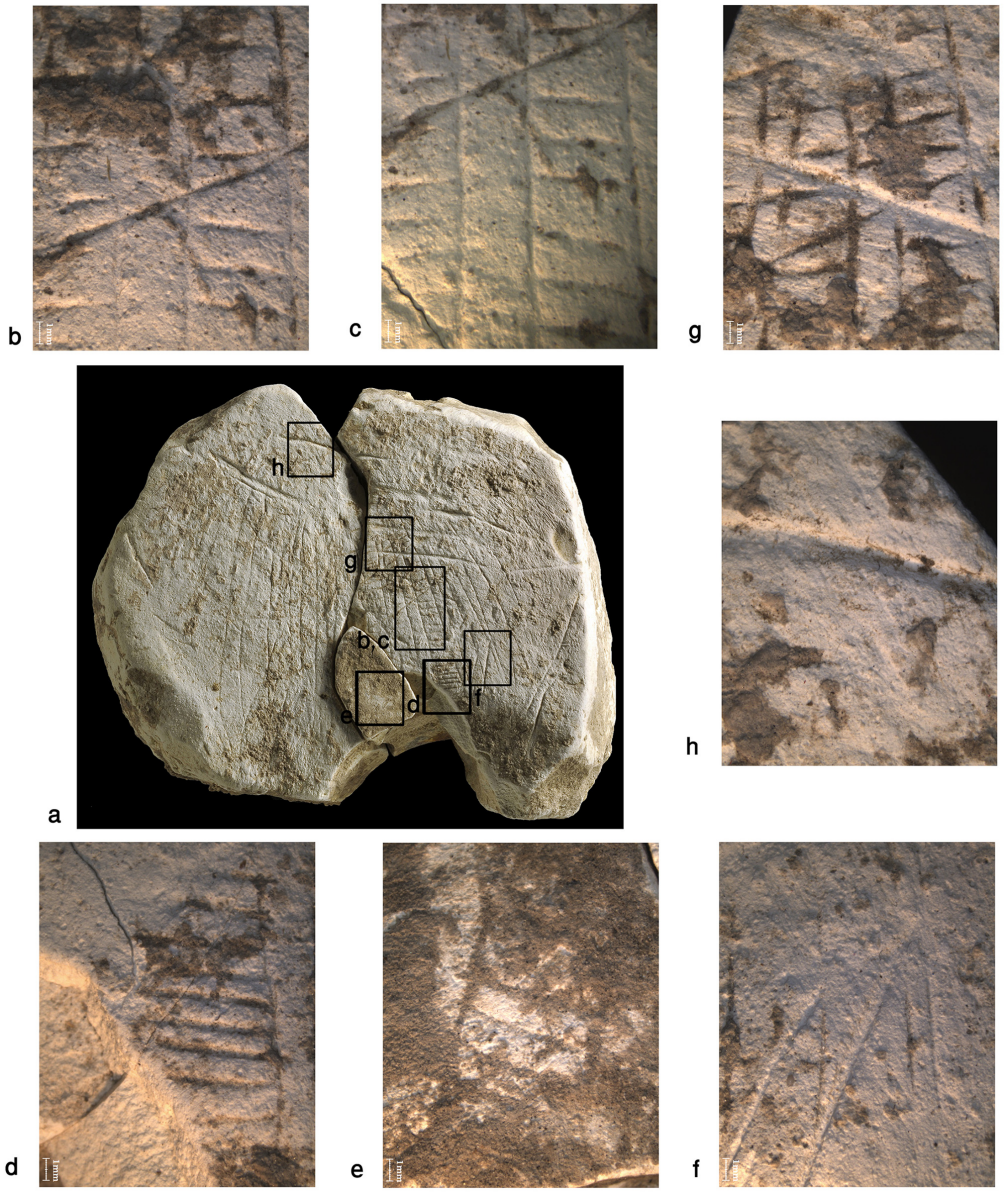
Details of the ladder pattern observed through the stereoscope. (a) Ladder motif; indicated are the localities enlarged on b-h. (b, c) Narrow line crossing the central and the right ladders. (d) Lower part of the right ladder. (e) Lower part of the left ladder. (f) "Cues". g) Broad line crossing the narrow lines and the ladders below them. (h) Broad line at the upper part of the plaquette. The scale of the enlarged fragments is 1mm.

The recto contains a motif composed of numerous extended parallel lines and three "ladders" which appear in the middle of the composition that is severely damaged by the breakage (here and afterwards we relate to the pattern conform its position as illustrated in Figs 13–15). The "ladders" seem to slightly taper in their upper part and are a little curved, following the contour of the round plaquette. The entire composition seems to broaden downwards as if the goal was to follow the shape of the plaquette, thus maximizing available space. The verso of the plaquette contains a crosshatched design. On one half of the plaquette the pattern is more prominent and better preserved than on the other. A red stain, associated mainly with the better preserved crosshatched pattern, is notable. The stain presumably represents ochre, although no chemical analysis has been undertaken yet to determine its nature.

The poor preservation of this item, the softness of the material and the fragility of the rather shallow pattern, virtually deteriorating with each single touch, made it really difficult to examine the engraving. The dark incrustations filling the incisions constituted yet another difficulty to overcome. Therefore, our observations made with the aid of stereoscope and scanning electron microscope are preliminary in essence. Yet, based on these observations several distinctive components and their interrelation could be distinguished.

**Fig 16:**
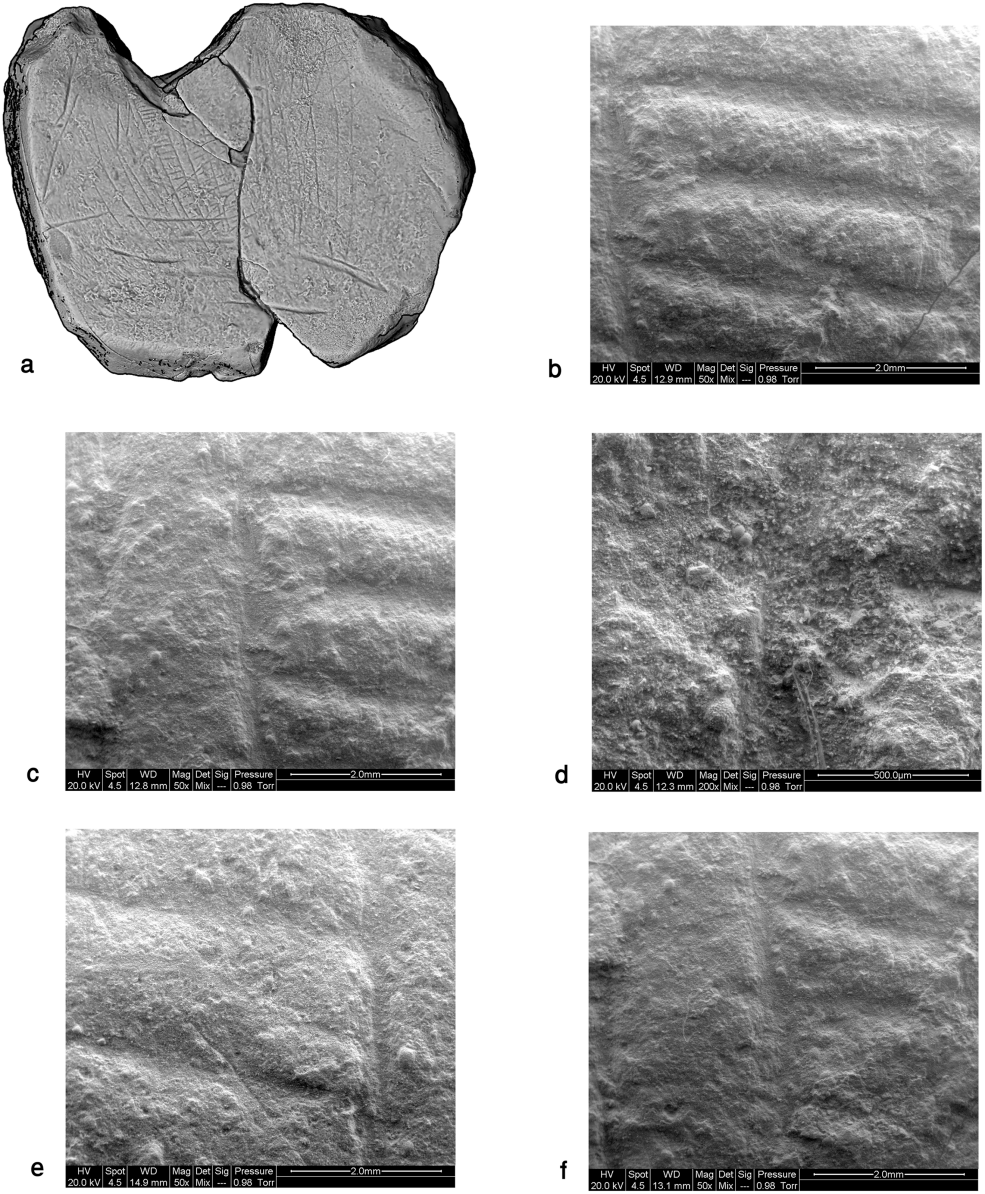
Details of the ladder pattern observed through SEM. Notice the orientation of the plaquette.

1. Three "ladders" incorporating the “rungs"–rows of short horizontal roughly parallel incisions and vertical “poles”–extended parallel vertical lines delineating the rows of "rungs" in the middle of the composition (Fig 14b). Not all the "rungs" are restricted to the space between the poles: some are longer than others, even extending significantly beyond the ladder's poles. Moreover, in each ladder the length, width and space between the "rungs" do vary, possibly indicating use of different tools or different working edges/angles of the same tool (Fig 15). In addition, it appears that the "poles" are narrower than the "rungs" (Figs 15 and 16). Intriguingly, some intersections indicate a possibility that the "rungs" were created prior to the "poles" (Fig 16). The "ladders" are roughly parallel to extended, vertical lines distributed over the entire surface of the plaquette. Some of these appear to be arranged in pairs, comprising "double lines" ([23]: p. 276) to the left and to the right of the central ladders. Moreover, there is some indication for several almost erased rungs associated with the "double lines". In other words, there is a possibility of (the once existence of) additional ladders, either removed intentionally, erased over time while the plaquette was in usage, or as a result of post-depositional processes.
2. Short oblique lines attached to some of the bands and seen mostly on the right side of the composition (Figs 14c and 15). These components are identical to “additional visual-kinesthetic and spatial elements or cues that were involved in giving meaning to the sum of the composition” observed on European specimens ([22]: p.447; see also [23]: p.287; [20]: p. 73). For the EQS plaquettes these "cues" are of further importance since identical short obliquely lines attached to the lower part of bird's feathers indicate a connection between the engravings, iconographical, purely stylistic or technical in nature, uniting all the three plaquettes into a single, coherent assemblage.
3. Long oblique lines crossing the "potential ladders", as well as the extended, parallel lines mentioned supra (Fig 14d).
4. Narrow lines crossing over the ladder motif in its upper part (Figs 14e and 15b and 15c).
5. Broad lines crossing both the ladder motif and the lines mentioned supra (Figs 14f and 15g and 15h).

## Discussion of the Engraved Motifs

The EQS plaquettes show variability in displayed imagery, incorporating both figurative and non-figurative motifs, in kind and treatment of the blanks themselves as well as in technical details of the engravings. At the same time, the common features observed on the engravings show that the plaquettes form a single coherent assemblage. Before venturing on an interpretation of the engravings, the patterning or structuring of the assemblage as such should be pointed out. Thus, the bird image and the chevron motifs, both created by rather broad, deep and uniformly looking incisions were displayed on small flakes or plaquettes without signs of significant preparation prior to the application of the engravings. In contrast, the ladder pattern engraved with mostly thin and shallow lines appears on a relatively large and carefully shaped and smoothed plate. These two characteristics, viz., variability and patterning of the motifs are taken into consideration in the interpretation of the images together with the conceptions developed in studies of local and European parallels and a selection of ethnographic examples.

Images of birds depicted on mobile items as well as on cave walls are known from European UP contexts (see [37] for a detailed review) and ethnographic records, in scenes representing ritual or cosmological belief systems or perceptions. To give some examples: a man with a bird-like head lying next to a “staff” with a bird mounted on top of it, from the Lascaux cave, has been interpreted as a shaman in a state of trance wearing a mask [38]. Birds represented on cave walls in Malakula, Melanesia are believed to embody spirit-helpers or to be an expression of experience of trance or weightlessness [39]. Pendants depicting selected aspects of seabirds’ anatomy such as feathers, webbed feet and bodies in flight created by the indigenous Beothuk of Newfoundland in Canada, reflect their belief that these birds were spiritual messengers capable of navigating through sky and over sea to the elated island of afterlife [40]. The EQS bird plaquette is too small to be displayed in a public ritual, yet, a spirit-related interpretation is plausible, especially considering the context of the find. EQS is a large, spatially well-organized site located in close proximity to a fresh water source in an ecotone conducive for hunting and foraging. These features, alongside the thickness of the archeological deposits and the absolute ages obtained for the two areas where the plaquettes were found conform to occupation for aggregation purposes over a long period of time. Aggregation of hunters-gatherers belonging to the same community implies regulating social issues related to reproduction, sharing resources and enhancing and/or renewing alliances—activities which are likely to be accompanied by rituals (see [41] and references therein). Moreover, aggregation of different groups implies the need for an image of group affiliation, an emblem (see e.g. [42] for applying the term). Thus, the interpretation of the bird plaquette as an object employed in ritual, or depicting ritual-related accessories, as well as interpretation in terms of some cosmological belief/perception or an emblem of a particular group of hunters are all viable.

A different interpretation, namely as cues or symbols of seasonal transformations observed in nature, was given to bird images identified as migratory species depicted on mobile items and on cave walls from European UP contexts [43]. This interpretation reflects the approach to Paleolithic art in terms of gathering information from the natural environment in order to indicate location and state of potential resources. This approach [43] implies that the visually conveyed information was shared amongst group members as well as that the group's children were taught to use this information properly. The identification of the EQS bird as a migratory species of bald ibis, provided independently by two ornithologists, allows an interpretation of the image along these lines. The bald ibis, overall black with red bald head, beak and legs, is a visually very prominent species and a perfect candidate to be referred to as a cue to some seasonal change and, possibly, to particular activities the group was engaged in while this seasonal transformation was approaching or actually taking place. Presenting a bird as an indicator for some phenomenon which otherwise lacks direct visual imagery on its own account points to the use of symbols, i.e., conventional signs whose meaning was understood by the other members of the group. Indeed, the knowledge how to create and read these signs properly is expected to be passed on through teaching the group's children [43].

The geometric pattern on the recto of the bird plaquette can be explained in a variety of ways, all in accordance with the different interpretations of the bird image presented above. Assuming a transcendental ritual was involved, the design could then be interpreted as an entoptic phenomenon—a variety of geometric signs seen in an altered state of consciousness caused by hallucination or otherwise [44]. Conceiving the image of a migratory bird as a signal to seasonal changes in nature correlates well with the interpretation of rhomb-like signs and patterns being symbols of a particular season, viz., autumn or summer ([43]: fig. 40b; [45]; see also [23]: p. 272). Moreover, a purely visual association of the chevrons as wedge-like renditions of flocks of migratory birds seen in the sky would also hints at seasonality, as this event occurs in the Levant in autumn and in spring. Accordingly, the roughly straight line along the margin can be interpreted as an abstracted rendition of a horizon whereas the slightly curved line above the bird’s head may represent the vaulted sky. While the analysis of the EQS faunal assemblage is still ongoing, the rich evidence from the roughly contemporary site of Ohalo II in the meantime strongly suggests that the late Pleistocene foragers in the Levant were aware of birds migration and that they were appreciative of their dietary value, as well as of decorative and other benefits of the different species [46].

Notably, the signs and combinations observed on the verso of the bird plaquette are repeated on the chevron plaquette. These include V-shaped incisions, some comprising rhomb-like figures, while others cross each other at the point of convergence. It further includes the line engraved along the plaquette’s edge, adjacent to the chevron motif. Reoccurring, identical signs on the two distinct plaquettes indicate intention and suggest that each component of the composition might comprise certain meaningful symbols, whereas the association between them and the whole pattern may contain some narrative or information (for parallels see [23]: p. 276–277).

**Fig 17:**
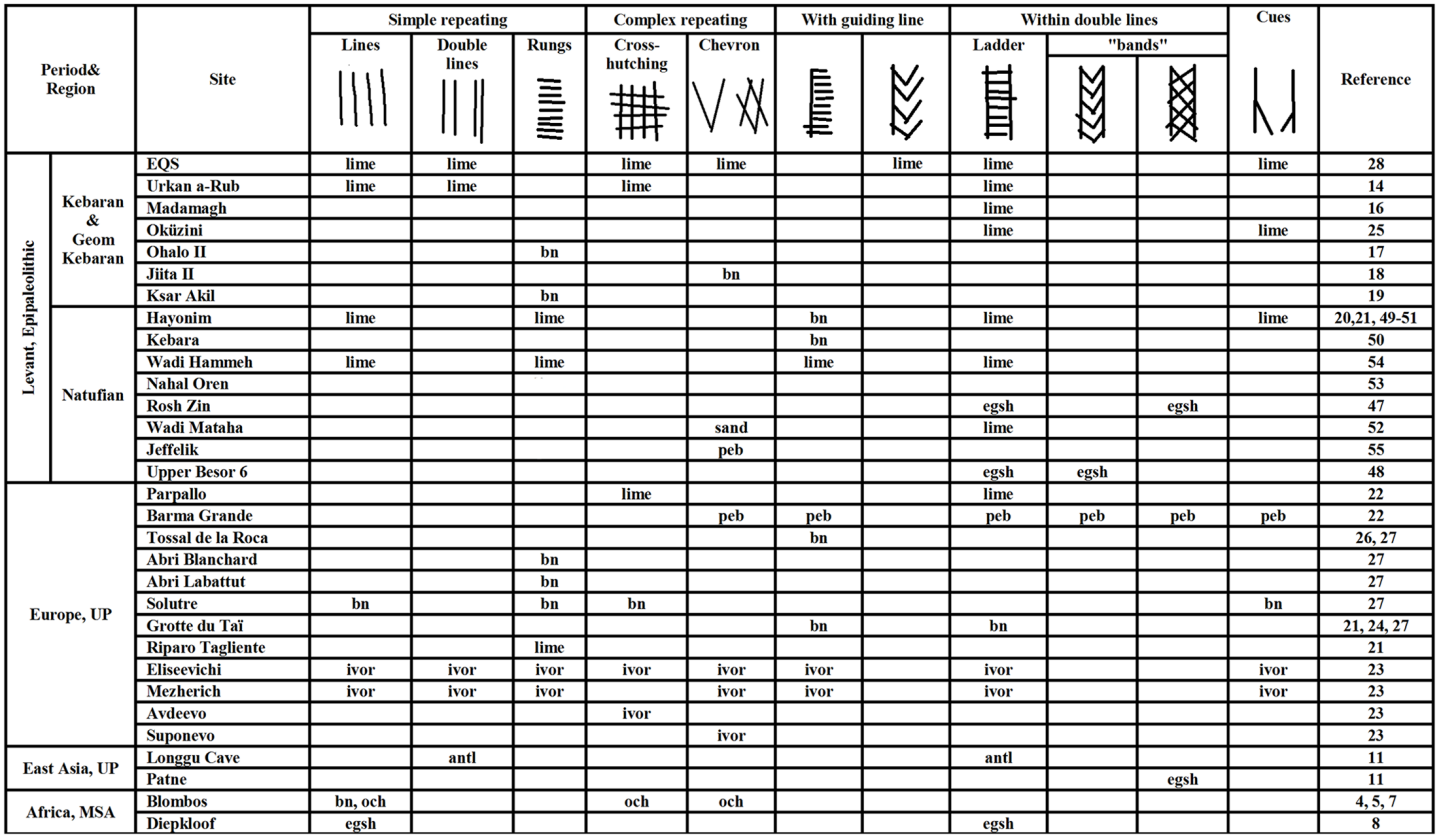
Signs and motifs present on EQS engravings and examples of similar designs found in contexts of European and Asian UP and African MS. lime: limestone; bn: bone; sand: sandstone; peb: pebble; egsh: eggshell; ivor: ivory; antl: antler; och: ochre (see [3] for examples of similar signs and motifs in parietal contexts in Europe).

The analysis of the ladder plaquette further suggests the use of conventional signs, but of principally different nature. The ladder pattern depicted on the recto of the plaquette resembles that found on a number of Levantine and European examples identified as "systems of notation", possibly concerning time and location for particular activities, events of aggregation, either for specialized hunting, marital issues, rituals, exchange of resources etc. [20–25] or as "artificial memory systems" (AMS) i.e., devices bearing encoded information ([26, 27], see Fig 17 for examples). Investigation of ethnographic devises of that kind [27] shows a variability in materials applied in their production (e.g., rope, shell, bone, wood), elements types (beads, ties, incisions, notches) and external/visual appearance (rosary, tally-stick, shell chains, suspended cords). However, the principals underlying the organization of the coded information can be universally summarized as being based on one to four major factors: morphology of the elements, spatial distribution of the elements, accumulation of the elements through time and number of elements involved [27]. Not all AMS possibly used by Paleolithic foragers would have been preserved in the archaeological record since they could have been applied to perishable materials. Therefore, engraved items, especially those containing repetitive designs such as the ladder motif, comprise the best candidates for investigation as possible devices bearing encoded information. Our analysis of the ladder pattern is preliminary and a more detailed study aiming at the determination of the morphology of the incisions, their orientation and sequence is still required. Yet, the observed variances in the "rungs‴ width, length and spacing suggest a real possibility that the conceived patterns were created on different occasions, i.e., contain a code based on accumulation through time. The presence of "poles", "cues" and sets of narrow and broad lines—elements apparently engraved following the "rungs‴ rows—may further support such a possibility. Alternatively, each row of "rungs" could have comprised an independent morphological feature created at one occasion (possibly through changing the tool's edge or angle). In such a scenario, the code would be based on three features, namely the morphology of the components—rows of "rungs", "cues", "poles", their number and spatial distribution. Moreover, the disposition of the morphologically similar elements–"rungs", "poles", and "cues"—rises a possibility of hierarchical organization within the code, providing a higher degree of flexibility, i.e., more options to encode information. In this regard it should be noted that the parallels showing the ladder motif from European and Levantine records (Fig 17) exhibit a variability with regard to the number and arrangement of the rows of "rungs‴, as well as in respect to their relation with other elements observed on the engravings.

The recovering or reading of the encoded information, possibly executed by a distinctive member of the community, the initiated person or the bard, could have been performed either visually or tactually [27]. In this respect the relatively large size of the EQS ladder plaquette and the smoothing of its surface are also of importance, as these characteristics corroborate the need of tactual or visual assessment of the pattern in all its details. Furthermore, it seems not unlikely that smoothing of the plaquette could have been performed each time the notations had filled out the whole surface, and the need for a blank surface (*tabula rasa*) arose. In this regard the circumstances of the plaquette’s discovery are also telling: it was found in situ, broken but in articulation, i.e., held together, suggesting discard of this artefact because of the crack (otherwise it would not have been found still in articulation), that is to say because the encoded information, whether built up through accumulation over time or based on morphology, number of the components and their spatial distribution became "unreadable".

The images and motifs observed on the EQS plaquettes reoccur in the rich repertoire of imagery characterizing the final Epipaleolithic Natufian (see [47–56], Fig 17)—a culture defined as being on the threshold to a Neolithic, agricultural, sedentary way of life [12, 13]. The artistic explosion associated with the social and economic transformations in that period was apparently accompanied by increased variability in the function or the intrinsic meaning of the graphic expressions applied. Evidently, notations or records in the form of ladder patterns continued to be used (e.g. [20, 21, 50, 54]) though the content or the stored information in the new, sedentary subsistence realm must have changed. Thus, one of the most prominent examples of such a notation—the large limestone slab deriving from Hayonim Cave—has been interpreted as a calendar possibly recording the start and amount of the winter rains, the growing of different cereals, preparation of inventory of harvesting and storage, or rituals related to these events and activities [20, 21]. Again, such an interpretation presupposes the presence of a specialist in record maintenance, a shaman, a community elder or the chief of a kinship group.

The diminutive size of some of the engraved objects found in a variety of Natufian sites have led authors to suggest conceptually different interpretations, attaching importance to the pattern itself rather than to its functional aspect. The patterns could comprise an emblem "adopted" by a particular group or family for successful survival in conditions of increased social complexity [49]; or, alternatively, these could have been produced and possessed by individual community members as an expression of connectivity with a broader cultural realm [52]. Moreover, in some cases the hidden, if not altogether secretive context of the images, obviously not intended for actual display, suggests the possibility that the very act of engraving and possessing knowledge of the act were of prime importance, rather than the actual visibility of the end product [56].

Geometric signs and motifs appear in Natufian contexts alongside numerous figurative imagery that includes zoomorphic representations of a realistic nature [49, 53, 57, 58]. Amongst these are one of the hallmarks of Natufian art—bone sickle handles carved with heads of ungulates—animals perennially present in the Southern Levant. In this regard it is intriguing to note that the images of the birds known from Natufian contexts constitute non-realistic, but highly stylized "avian" bone pendant [54], i.e., items representing an idea of a bird in flight rather than a particular species actually existent in nature. In this respect Natufian birds notably differ from the schematic but still easily recognizable seasonal species depicted on the EQS plaquette. Certainly, seasonality must have been important for Natufian foragers who started experimenting with plant resources alongside intensive hunting. Yet, it is well possible that particular season-related activities became less important. Accordingly, visually conveyed signs symbolizing these specific activities may have become less relevant and over time have ceased altogether.

Certainly, the full meaning or purpose of the ladder, chevron and crosshatching designs as well as bird images created by different Epipaleolithic groups will most probably remain enigmatic to us. Yet, the obvious dichotomy in frequency of imagery found in contexts of Epipaleolithic nomadic hunters compared to complex Natufian foragers emphasizes the deep connection between the use of graphic symbols and social and subsistence realms [10]. This association can further explain the paucity of imagery in pre-Natufian contexts in the Levant in comparison with their roughly contemporary European counterparts. In late Pleistocene Europe—a region defined by clear seasonality—the availability of different resources was fixed in time and related to seasonality and hence cyclic. Therefore it was crucial for bands to be prepared ahead of time for group hunting, fishing, collecting plants etc. In such conditions seasonal aggregation of mobile groups—an activity requiring keeping a calendar or schedule related to time and location of such events—would be an efficient strategy [21]. In the Levant where seasonality is less prominent, the availability of different kinds of resources is relatively stable throughout the year. In particular, the main species of wild game, i.e., gazelle, fallow deer and wild cattle are not migrating but staying in their habitats thus remaining largely available for hunting on a perennial basis. Such a situation implies reduced need in aggregation and, subsequently, in keeping track records or "calendars"/schedules of groups meetings. The EQS plaquettes—the rare evidence of graphic symbols applied by late Pleistocene hunters-gatherers in the Levant—reflect the function of this site as a locality of group aggregation within a logistical system of mobility and hence indicates a social complexity of pre-Natufian foragers [14, 59].

The similarity in use of graphic images applied by Late Pleistocene hunter-gatherers in distinctly remote areas (such as, e.g., Levant and Europe) is an intriguing issue addressed in the past by several authors (e.g., [14, 52, 60, 61], Fig 17, see also [3] for review concerning similar signs and motifs in parietal contexts in Europe). While convergent development is indeed a plausible explanation, other possibilities should also be considered. An alternative explanation could be interaction, albeit not necessarily a direct one, between remote mobile groups. Such an interpretation is not inconceivable, considering the distribution of several very close parallels, i.e., EQS, Urkan a-Rub [14], Oküzini Cave [25] Parpallo and Barma Grande [22] across the Mediterranean basin. Another possible explanation for the presence of similarly looking notations in areas considerably distanced from one another, would be to assume a common origin of this symbolic conduct. Creating complex notations or storing concepts with the aid of tangible, graphic symbols, has been defined as a "fundamental turning point in the evolution of human cognitive abilities and cultural transmission" [6]. The variability and complexity of the earliest examples of notational systems found in European contexts suggest that this behavior has a much earlier origin, possibly traceable to African contexts pre-dating the migration of modern humans out of that continent [6]. All the signs and motifs appearing on the EQS plaquettes as well as on other Levantine, European and East Asian examples dated to the late Pleistocene, i.e., ladders, crosshatching and chevrons, have been reported over the last few decades in association with MSA contexts in South Africa (e.g. [4–9], Fig 17) as well, making such a scenario all the more plausible.

## Conclusions

The singular, figurative bird image and the repetitive, non-figurative patterns incised on three plaquettes from EQS show complex and well-structured use of symbols. The quantity and variability of the motifs make the assemblage unique for the late Pleistocene Levantine record. Based on the parallels from roughly contemporary European contexts of nomadic hunters as well as from the local Natufian sites of sedentary foragers, EQS engravings can be understood as records or notations related to availability of resources and timing of aggregation events. Such an interpretation suggests that the site served as a place for seasonal meetings of nomadic groups and implies social complexity of pre-Natufian foragers. Interpretation of graphic imagery in terms of social and subsistence needs might be a valid tool to explain the varying frequencies of relevant artefacts in different and remote regions of the Late Pleistocene word. At the same time, the similarity in image use sustained by Late Pleistocene foragers in different environmental and social settings supports the possibility that symbol-mediated behavior might have a common and much earlier origin.
